# Overview of Antibody Drug Delivery

**DOI:** 10.3390/pharmaceutics10030083

**Published:** 2018-07-04

**Authors:** Sahar Awwad, Ukrit Angkawinitwong

**Affiliations:** 1 UCL School of Pharmacy, London WC1N 1AX, UK; ukrit.angkawinitwong.11@ucl.ac.uk; 2 National Institute for Health Research (NIHR) Biomedical Research Centre at Moorfields Eye Hospital NHS Foundation Trust and UCL Institute of Ophthalmology, London EC1 V9EL, UK

**Keywords:** antibodies, protein, pharmacokinetics, drug delivery, stability

## Abstract

Monoclonal antibodies (mAbs) are one of the most important classes of therapeutic proteins, which are used to treat a wide number of diseases (e.g., oncology, inflammation and autoimmune diseases). Monoclonal antibody technologies are continuing to evolve to develop medicines with increasingly improved safety profiles, with the identification of new drug targets being one key barrier for new antibody development. There are many opportunities for developing antibody formulations for better patient compliance, cost savings and lifecycle management, e.g., subcutaneous formulations. However, mAb-based medicines also have limitations that impact their clinical use; the most prominent challenges are their short pharmacokinetic properties and stability issues during manufacturing, transport and storage that can lead to aggregation and protein denaturation. The development of long acting protein formulations must maintain protein stability and be able to deliver a large enough dose over a prolonged period. Many strategies are being pursued to improve the formulation and dosage forms of antibodies to improve efficacy and to increase the range of applications for the clinical use of mAbs.

## 1. Introduction

Monoclonal antibodies (mAbs) are by far the largest class of therapeutic proteins and a key driver in the biopharmaceutical growth [1]. Currently, humanized mAbs are the fastest growing group in clinical trials [2]. Most therapeutics mAbs that are available share common functions such as blocking target receptors or ligands, thus decreasing the overall activity of certain pathway. The Food and Drug Administration (FDA) approved the first mAb in 1986 (Orthoclone OKT3, muromonab-CD3) for the prevention of kidney transplant rejection [2,3] (later withdrawn) and many more antibodies and antibody derivatives (e.g., fragment antigen-binding (Fab), fragment crystallisable region (Fc)-fusion proteins, etc.) [4] have been developed since [5]. A majority of mAb therapeutics have been approved for oncology, rheumatoid and autoimmune diseases [4]. mAbs have revolutionized the treatment of ophthalmic conditions (e.g., use of anti-vascular endothelial growth factor (VEGF) medicines such as ranibizumab (Lucentis^®^) in the treatment of wet age-related macular degeneration, AMD) [6]. Monoclonal antibodies exhibit good safety profiles and enhanced efficacy; and have been reported to show higher success rates in progression through early clinical development [7]. Approximately 70 mAb products have been predicted to be available in market by 2020 for the treatment of various diseases [8].

**Figure 1:**
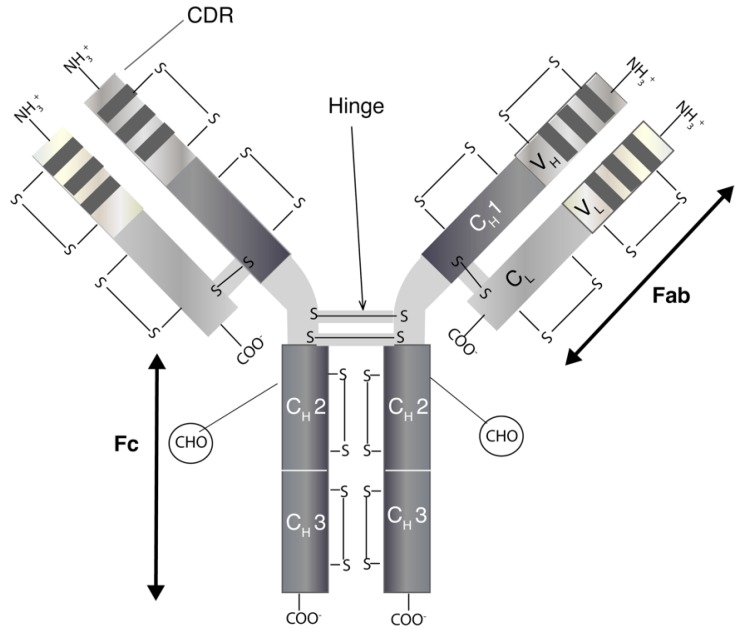
A schematic diagram representing the modular structure of a monoclonal antibody (mAb). (Abbreviation: CDR: complementarity-determining region; COO-: carboxy terminal; CH: constant domain, heavy chain, CL: constant domain, light chain; Fab: fragment antigen-binding; Fc: fragment crystallisable region, NH3: amino terminal end, S-S: disulfide bond; VH: variable domain, heavy chain; VL: variable domain, light chain)

Monoclonal antibodies are immunoglobulins (Ig) of which there are five classes (IgA, IgD, IgE, IgG and IgM) [9]. The most relevant for therapeutics is IgG, which has four subclasses (IgG1, IgG2, IgG3 and IgG4) [10]. IgGs are bivalent molecules with a molecular weight of ~150 kDa, which can be highly site specific with affinities ranging from nano- to picomolar. IgGs (Figure 1) have two separate identical heavy (H) and light (L) chains [11]. The N-terminal domain of an IgG consists of a variable (V) region with the complementarity-determining region (CDR) that binds to a specific epitope on antigens. Other domains within the IgG make up the constant (C) regions. The structure of an IgG is broadly divided into the Fab (2/3) and Fc regions (1/3). The two identical Fabs each comprise of a light chain that is closely associated by non-covalent interactions with the heavy chain. There is a solvent-accessible interchain disulfide (S-S) between the light chain and heavy chain of the Fab. The heavy chains are closely associated through non-covalent interactions within the Fc region. The hinge region links the Fabs to the Fc and there are (usually) two solvent accessible disulfides between the heavy chains in the hinge region. There are also intra-molecular disulfides within each constant and variable region of both the heavy and light chains. These disulfides are not solvent-accessible. Many aspects of mAbs, including epitope specificity, immunogenicity, pharmacokinetic and immune-related effector functions, are active areas of research that is focused on the continued evolution of antibodies for therapeutic use [12].

The Fab structure allows different IgG molecules to identify different antigens [13]. Porter and Edelman (1972) won the Noble Prize for elucidating the structure of antibodies by proteolytic digestion using thiol proteases, e.g., papain. With the use of papain, Porter was able to cleave the IgG molecule into two Fabs and an Fc. The Fab can bind to a specific antigen while the Fc is unable to block binding to a specific antigen [14,15]. Fabs have been used in the determination of antibody–antigen interactions [16]. Fabs usually have better tissue penetration than full length IgGs and can allow interactions with enzyme sites (which IgGs can find difficult to access). However, Fabs do display fast off-rates and poor retention times compared to IgGs on target as a result of the monovalency of Fab [17]. The FDA and the European Medicines Agency (EMA) have approved three Fabs i.e., certolizumab pegol (Cimzia^®^), ranibizumab (Lucentis^®^) and abciximab (Reopro^®^). Ranibizumab is a humanized Fab with specificity to all isoforms of VEGF [18], and has demonstrated good signs of biological activity and acceptable safety when administered as an intravitreal injection up to 6 months in patients with neovascular AMD [19,20,21]. Polyclonal Fabs have also been marketed such as crotalidae polyvalent immune Fab (ovine) (CroFab^®^), digoxin immune Fab (DigiFab^®^) and digoxin immune Fab (ovine) (Digibind^®^) [22]. The biological function of approved Fabs is restricted to the monovalent binding of the Fab to its target. Fabs conjugated to exogenous functional moieties have been in clinical development including twelve Fabs conjugated to cellular toxins, seven to radioisotopes, three to cytokines and one Fab conjugated to an enzyme targeting toxic metabolites. Fabs can be produced by antibody digestion using proteolytic enzymes or by direct expression from bacteria, mammalian cells or yeast [17]. 

The Fc domain can bind with Fcγ receptors (FcγR) to cause effector functions. The FcγR are largely expressed on immune cells such as natural killer (NK) cells, macrophages and dendritic cells [23]. The Fc domain endows mAbs with effector functions involving the stimulation of a specific immune response to eliminate the mAb target molecule complex. This can include the release of cytotoxic granules and apoptosis as a result of engagement of FcγRIIIa on NK cells (antibody-dependent cell-mediated cytotoxicity, ADCC); the phagocytosis of antibody-coated target molecules by macrophages by engaging FcγRIIa (antibody-dependent cell-mediated phagocytosis, ADCP) and the engagement of complement protein C1q (complement-dependent cytotoxicity, CDC) [7,24,25]. 

Cetuximab, an epidermal growth factor receptor (EGFR) inhibitor, binds to antigen on the surface of tumor cells and exhibits its anti-tumor activity through ADCC [26]. These effector functions can have therapeutic benefits, but may also cause adverse effects in some diseases. For example, it was found that the anti-cancer agents trastuzumab and rituximab failed to prevent tumor growth in Fcγ-deficient mice [27], suggesting the significance of effector functions on the efficacy of mAbs. The ADCC and CDC functions of rituximab and alemtuzumab can contribute to cell lysis and unintended cytokine release [28]. Also, the Fcγ binding properties vary among the IgG subclasses. For example, IgG1 subclass can bind to all FcγR, whereas IgG4 weakly binds to FcγRIIb and moderately to FcγRI [29]. Therefore, rational design of both the Fab and Fc domains is essential to modulate therapeutic efficacy and safety profile of mAbs.

**Figure 2:**
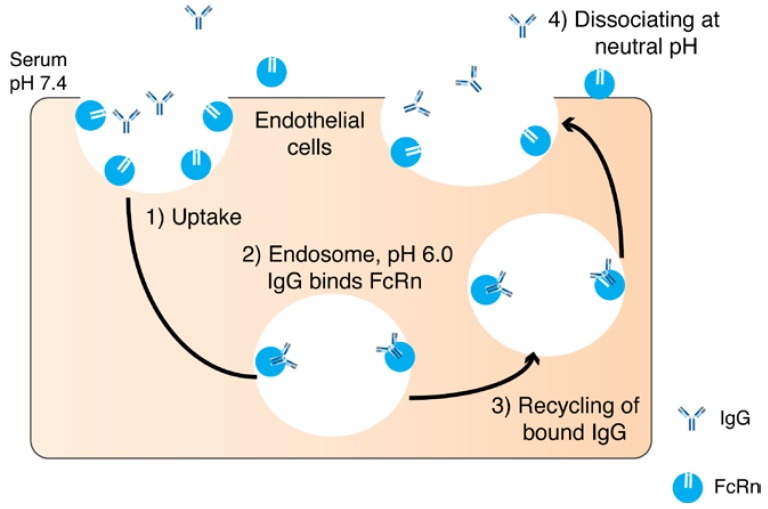
A schematic diagram illustrating neonatal Fc receptor (FcRn) recycling pathway: (**1**) Immunoglobulin G (IgGs) are internaliszed into endocytic vesicles. (**2**) Endosome becomes acidic resulting in the binding of Fc domain to FcRn. (**3**) Bound IgG are recycled back to the cell membrane and (**4**) IgG dissociates at neutral pH (7.4) from FcRn and is released back into the blood.

Another function of the Fc is to recycle the antibody while it circulates in the blood. The specific transport of IgG from mother to offspring through the placenta is carried out by the neonatal Fc receptor (FcRn) [30,31]. FcRn is a histocompatibility class I-related protein that interacts with IgG and albumin in a pH dependent manner [32]. In rodents, the acidic pH (pH 6.5) allows for high binding between the Fc portion of the IgG and FcRn. FcRn transcytoses IgG after binding and releases on the neonatal side at neutral pH (pH 7.4). In humans, the IgG transfer occurs across the syncytiotrophoblast of the placenta. The FcRn binds to the maternal IgG after endosome acidification and releases the IgG in the fetal circulation at physiological pH [30] (Figure 2). The inhibition of FcRn up-regulation or the FcRn elimination pathway results in an increased elimination and prolongation of the half-life of an IgG [33] FcRn transports its ligands across a cellular monolayer providing a route for proteins into the blood stream (across the epithelial barriers) [34]. FcRn has been found in mucosal sites in adult mammalians and a number of studies have discussed the role of FcRn-mediated IgG in the intestine and gastrointestinal tract (GIT) [34,35,36,37,38,39]. The role of FcRn has also been described in pulmonary drug delivery [34,40,41], where the quantity and quality of IgG in the airways are regulated by FcRn [40]. However, FcRn recycling has been reported not to contribute to the vitreal half-life of an IgG when injected into the back of the eye [42]. The expression level of FcRn in the eye and the systemic epithelium might contribute to the FcRn not playing a major role in the eye when compared to the central compartment [43].

The development of therapeutic mAbs involves several complex processes including mAb screening, engineering, production and purification. The productivity of each step has been improved as technology has evolved in recombinant techniques, bioprocesses and affinity-based purification. Only mAb screening and engineering will be discussed here; more comprehensive reviews of upstream and downstream mAb production are detailed elsewhere [44,45,46,47]. 

Generally, mAbs are generated by means of animal immunization as a part of screening process. Hybridomas are engineered cells that are capable of producing mAbs [48] and were developed by George Kohler and Cesar Milstein who won a Nobel Prize for their discovery. The main advantage of hybridoma technology is its ability to grow continuously as well as its ability to produce a large amount of pure antibody [48,49]. The original hybridoma technology was established more than 35 years ago, which involved the use of hemagglutinating virus of Japan (HVJ) or Sendai and poly(ethylene glycol) (PEG) for fusing antigen-sensitized B lymphocytes and myeloma cells [50]. 

Briefly, animals (usually mice or rats) are immunized by injection with a soluble antigen that is mixed with an adjuvant. The antigen-specific producing plasma cells from the spleen are then isolated and are fused with a cancerous immune cell (called a myeloma cell) using fusing reagents such as HVJ or PEG [50]. The fused cells are then cultured in a growth medium supplemented with hypoxanthine-aminopterin-thymidine (HAT) medium. The cells that survive are the ones that express hypoxanthine guanine phosphoribosyl transferase (HGPRT) and are called hybridoma cells. Hybridoma cells become visible after 4 days and are grown for about 3 weeks. The culture supernatant is eventually screened for secretion of the desired antibody (assays include Western blot and enzyme-linked immunosorbent assay (ELISA)) [50]. Given the rapid proliferation of myeloma cells and the production of specific antibodies, these two properties lead to the feasibility to produce mAbs in vitro. Plasma and myeloma cells can also be fused using an electrical field (pearl-chain formation) or laser radiation in order to enhance fusion efficiency [50].

Despite the advance of hybridoma methods, the technique encounters challenges such as low yield and contamination [51]. Performing under controlled in vitro conditions, the combination of genetic engineering and in vitro selection strategy provides more scalable and high-throughput mAbs production that is suitable for commercial development [52]. Phage display is a widely used in vitro selection for screening therapeutic mAbs [53]. Adalimumab, an anti-tumor necrosis factor (TNF) drug, was the first phage-display-derived mAb approved for clinical use [51]. 

The phage display technique can produce expressed antibody motifs, which are often Fab or single-chain variable fragments (scFv) [54]. The pool of the antibody phages creates an antibody library, which is used in the selection process or ‘panning’ step to screen the antibody candidates based on the binding property to target molecules. The selected phage will be re-amplified in *Escherichia coli* where the new antibody phage is generated. The panning steps are repeated for 2–3 rounds potentially increasing the number of antigen-specific antibody phage clones. The resulting antibody genes can be sub-cloned to produce other antibody formats such as scFv-Fc or IgG [53]. The advantage of mAbs obtained from phage display technique is the improved affinity in the picomolar range whereas the mAb affinity was reported to be approximately 100 pM by in vivo immunization [55].

**Figure 3:**
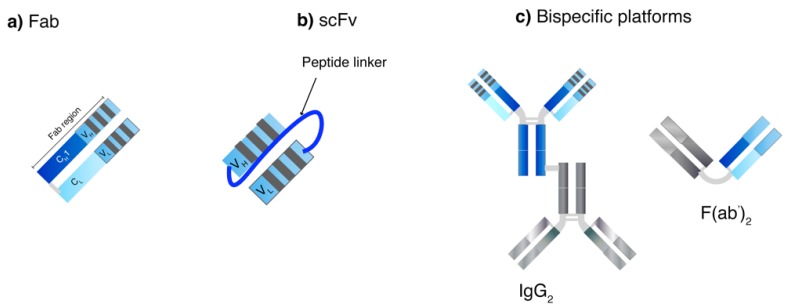
Small-mAb derived fragment technologies and next generation mAbs. (**a**) Recombinant fragment antigen-binding (Fab), (**b**) Single-chain variable fragment (scFv) and (**c**) bispecific mAb platforms. The latter can comprise two covalently linked heterogenous IgG (IgG_2_) or heterogenous Fab domains (F(ab’)_2_).

Apart from screening process, genetic engineering also plays a crucial role in producing human-mAb structures to reduce overall immunogenicity of generated mAbs from animals. Chimeric mAbs display approximately 60–70% human homology and are produced by combining the gene encoding murine Fv and human Fc [46]. A murine CDR can be grafted onto a human framework to generate humanized mAbs (90–95% human) [56]. Without the murine CDR, a fully human mAb (100% human) can also be generated. Moreover, genetic engineering has been examined to generate small mAb-derived fragments such as recombinant Fab, scFv and bispecific antibodies (Figure 3) [46]. Recombinant Fabs contain one heterodimer of CH1 and VH covalently linked with CL and VL domains while the Fc portion is deleted. ScFv technology uses one VH and VL sequence responsible for antigen domain binding domains connected with a linker sequence or bispecific mAbs, which allow dual targeting. They can be used to bind to various antigens (haptens, proteins and pathogens) and can be used alone or as fusions in ELISA [17]. ScFv are smaller in size as compared to Fabs resulting in better tissue penetration and pharmacokinetic profiles, however, they have also been reported to have fast off-rates and poor retention times [17]. The modification of the length of the scFv linker can create derivatized multivalent scFv such as bivalent scFv (diabodies) and trivalent scFv (tribodies) that enable multi-mAb binding [46]. In one study, a 5-fold increase in half-life and 30-fold increase in tumor uptake were observed with a site-specific polysialylated anti-carcinoembryonic antigen (CEA) scFv as compared to an unmodified scFv [57]. So far, no multivalent mAb product is commercially available.

Bispecific antibodies are intended to bind to different antigens or epitopes by combining the specificities of two antibodies [58]. One hope is to develop bispecific antibodies to address applications where they can exploit spatial–temporal relationships that are not possible by using a combination or mixture of antibodies. There are several potential advantages to bispecific antibodies: (i) they can redirect specific immune cells to the tumor cells to enhance tumor killing; (ii) they can enable simultaneous blocking of two different mediators/pathways that exert unique or overlapping functions in pathogenesis; and (iii) they can potentially increase binding specificity by interacting with two different cell-surface antigens instead of one. It is generally difficult to ensure multifunctional proteins such as bispecific antibodies are isolated in a pure and sufficient amount during early development and at a decent scale for production [59].

In essence, fully human mAbs can also be produced by performing a hybridoma approach in a transgenic mouse in which the murine Ig gene is knocked out and is replaced with a human-derived segment, thus enabling the production of entirely human mAbs [60]. This transgenic approach can avoid a human anti-mouse antibody (HAMA) response. Owing to their capabilities to produce fully human mAbs, phage-display and transgenic mice-derived mAbs gain relatively high success rate in the later stages of clinical trials [51].

## 2. General Limitations and Formulation Challenges

### 2.1. Pharmacokinetic Limitations

While most mAbs can display prolonged circulation times by FcRn-mediated recycling, many therapeutic proteins have short in vivo half-lives, which is usually a matter of hours to a few days [61]. For example, bevacizumab is a mAb that is used unlicensed for neovascularized ocular tissues and it is a cost-effective anti-VEGF medicine that is clinically equivalent to ranibizumab [62,63]. An intravitreal dose of bevacizumab (1.25 mg, 50 μL) and ranibizumab (0.5 mg, 50 μL) yields half-life values of 6.7–10.0 and 7.2–9.0 days respectively [64,65,66,67]. The short in vivo half-life results in frequent drug dosing, which can increase the chances of undesirable side effects [68] and can result in high costs and poorer compliance to both patients and health care systems [69]. In general, smaller modified antibodies (e.g., without the Fc) have shorter in vivo half-lives when administered via other routes [70].

Most mAbs are limited in their ability to penetrate and accumulate in tissues due to their large size. The solution size can have a direct impact on the pharmacokinetic properties and affect clinical efficacy. Therapeutic proteins can be passively distributed from the blood circulation to peripheral tissues by convective transport through fenestrae pores on capillary walls, or through the transcellular pathway via endothelial cells [71]. The latter mechanism highlights the possibility for active transport of mAbs, which can include cellular uptake of proteins in surrounding fluid (fluid-phase pinocytosis), receptor-mediated endocytosis (FcγR-mediated) and phagocytosis by immune cells [71]. In terms of mass transport, passive diffusion underpins the permeation or absorption of drugs across cell membranes and depends on the size, hydrophilicity–hydrophobicity and charge of the compounds. However, passive diffusion is limited for biotherapeutics owing to their large solution size and charge.

Given that subcutaneous administration is a desired route of administration, macromolecules are likely to be restricted to the interstitial space after injection owing to their large size [72]. In general, biotherapeutics can reach blood circulation by two pathways: via blood capillaries or lymphatic vessels. Absorption through blood capillaries relies on passive transport and is restricted to compounds with a molecular weight cut-off (MWCO) of up to 16 kDa [73]. Hence, most biotherapeutics will not be able to be transported via capillary routes, but rather rely on the lymphatic system where the protein reaches the systemic circulation at the thoracic duct [74]. The distribution of therapeutic proteins is limited to plasma rather than tissue [75]. As a consequence, delivering proteins to maintain effective therapeutic concentration at target tissues is challenging. For example, bevacizumab and ranibizumab are given intravitreally. Owing to the size of both anti-VEGF medicines, it is unlikely to deliver a significant level of both drugs to the posterior segment of the eye by systemic administration, where the blood retina barrier (BRB) is a major barrier for drug transport [76]. The subcutaneous administration of trastuzumab is enabled by the use of recombinant human hyaluronidase (rHuPH20) [77], which behaves as a permeation enhancer. Subcutaneous trastuzumab has a fixed dose of 600 mg administered 3-weekly and avoids weight-based infusion dosing [78]. rHuPH20 enhances the infusion rates and penetration of molecules up to 200 nm in diameter up to 20-fold without eliciting inflammation, vascular permeability, immune-genic or allergic reactions [79].

Enzymatic degradation, either at the site of administration or while in circulation, can further reduce the pharmacokinetics of most classes of proteins. Protein-based medicines usually exert their action extracellularly (e.g., cell surface receptor interaction and ligand binding). It is generally known that proteins and mAbs are prone to undergo enzymatic degradation and unfolding especially in the GIT. Oral delivery systems for these biologically active compounds are hence challenging to develop, unlike most small molecule drugs. Thus, the routes of administration of biologics are normally parenteral injection (intravenous, subcutaneous, intramuscular or intradermal). Most proteins and mAbs are now formulated for subcutaneous injection [74]. Even if parenteral application is used, macromolecule drugs can still suffer from pre-systemic degradation by enzymes such as proteases and hydrolase, since these are abundant throughout the body [80].

The elimination of mAb therapeutics can be accelerated by immunogenicity and the development of antibody-drug antibodies (ADAs), particularly with those proteins derived from animals. ADAs can also cause acute hypersensitivity or infusion reactions. Furthermore, ADAs can also competitively bind to the active region of the therapeutic protein such as the receptor-binding site to neutralize the antibody drug, therefore compromising efficacy. ADAs can also unpredictably change drug pharmacokinetic properties, biological effects and the toxicity profile [81]. Humanized and fully human mAbs and other therapeutic proteins are less immunogenic in humans compared with non-human derived proteins (e.g., murine antibodies), albeit humanized proteins could also cause an immune response in human beings [82]. Using animals to predict the human response to a candidate protein is another challenge in the development of protein therapeutics [83]. Animals develop an immune response against antigens of interest, however, the level of immunogenicity between animal models and humans is relatively different. It is reported that immunogenicity is over-estimated in conventional animal models making them unreliable to predict patient immunogenicity [84,85] owing to different mechanisms in the immune response between humans and animals [12]. Consequently, the immune responses obtained from animal studies are equivocal to predict clinical consequences (e.g., production of neutralizing antibodies and neo-epitopes on modified proteins) [85,86] and translate into human studies.

Like most classes of therapeutic proteins, antibody-based medicines generally tend to be cleared more quickly when used in a high dose as a result of possible aggregation [26]. However, this also depends on the precise clearance mechanism of the protein molecule. High protein dose will have a faster elimination if phagocytosis in the reticuloendothelial systems (RES) plays a major role. In contrast, the elimination rate will be decreased with high doses if endocytosis is the major elimination process [87]. As endocytosis is a receptor-mediated process, this suggests that the saturation of receptors can influence the bioavailability of protein drugs targeting surface receptors. This phenomenon is known as ‘antigen sink’ and is commonly found with mAbs targeting internalizing receptors [88]. For example, trastuzumab, an anti-human epidermal growth factor receptor (HER) 2 antibody, was eliminated slowly with a high dose [89]. As mentioned earlier in this section, a frequent dosing regimen is required to maintain the drug at therapeutic levels, which can have negative implications in the clinic. An infusion reaction is a common adverse effect associated with parenteral treatment. For example, a study showed that patients developed acute reactions with increasing doses of infliximab with 61% of patients experiencing an acute reaction at the fifth dose [90].

### 2.2. Formulation Challenges

Biological macromolecules including mAbs have three-dimensional (3D) structures known as the tertiary structure. An intricate balance of intra- and intermolecular interactions between amino acid functional groups and external environments dictates the folded structure. A range of non-covalent interactions is crucial to maintain the native folded structure, e.g., electrostatic interactions, van der Waals interactions of the backbone and side chain residues, hydrogen bonding and hydrophobic interactions [91]. As the folded structure is in dynamic equilibrium, any factors shifting the interaction balance can cause the structure to change, which can contribute to an unstable state of the large molecules. For example, in aqueous solution more soluble amino acid residues become exposed and interact with accessible solvent molecules while non-polar residues are encapsulated forming a hydrophobic core.

Protein folding is a function of the amino acid sequence and gives the biologically active form of the protein. However, protein in the native structure can be unfolded via an intermediate state or undergo direct unfolding to a denatured state. When the intermediate or unfolding species is formed, the variants are prone to assemble to form more stable complexes such as aggregation, owing to their higher free energy. Aggregates are composed of more than one monomer in any form and are strengthened by either covalent or non-covalent bonds. The type of monomer association can influence the reversibility of the aggregates; for example, aggregates of native monomer clusters can be dissociated by dilution [92]. Precipitation or irreversible aggregates may result from the nucleation of different monomers [93]. A number of stress factors can lead to aggregation, the most common ones in manufacturing being temperature, mechanical and freeze/thaw stress [26]. High temperatures can lead to conformational destabilization or partial/complete unfolding [26] and a change in pH can lead to aggregation. For example, a study compared the stability profiles of IgG1 and IgG4. IgG4 was reported to form more soluble aggregates than IgG1 at lower pH and high temperature due to reduced conformational stability from lower unfolding temperature and changes in tertiary structures [94].

As the tertiary structure of biologics are susceptible to physical stress from the environment, structural alteration of mAbs can emerge at any stage during the manufacturing process, from initial protein expression through processing to storage [95]. Structural transformation can be ascribed to the presence of non-physiological conditions during the processes that may drive the adaptation of structural variants in the finished product. It is a major issue and challenge in biopharmaceutical development that is distinctly different to the production of small molecule drugs where such problems do not arise. For instance, stresses such as buffer choice, manufacturing techniques and choice of containers can potentiate this issue [96]. A study reported the stability of a model murine IgG3 in various buffer solutions using ultraviolet/visible spectroscopy (UV-VIS) and size-exclusion chromatography (SEC). The use of acetate was reported to form visible precipitates, whereas arginine and histidine were shown to improve stability for a long-term storage and multiple freeze/thaw cycles of IgG3 [97]. A study demonstrated how lyophilization led to a pH shift in protein solutions resulting in a change in protein activity [98]. Adjusting the concentration of buffer salts could therefore help prevent activity changes. The pH of lyophilizate is also critical to control the aggregation of recombinant vaccine antigens and often additional stabilizers such as trehalose is required to maintain the integrity of the antigen [99].

Additionally, the presence of non-native proteins in any finished mAb-based biopharmaceutical products must be avoided. Aggregation can cause non-uniform dosing when drawing protein solutions from vials [100]. Unstable biologics can contribute to a lack of therapeutic effect or adverse effects. For example, the aggregation of IFN–β can stimulate the production of neutralizing anti-drug Abs (NAb), which blocks the IFN receptor binding, thus lowering clinical efficacy [101]. The unwanted effects can also arise from induced NAbs on the normal function of endogenous proteins, especially in the case of therapeutic hormones or cytokines. Notably, pure-red cell aplasia (PRCA), a sudden severe anemia, was developed in patients treated with Eprex^®^ rh erythropoietin [102]. The complication emerged as a result of NAb induced against Eprex^®^ blocking endogenous erythropoietin [103]. The NAb was induced by aggregation driven by the presence of formulation ingredients such as polysorbate 80 [104] and silicone oil in pre-filled syringes [105,106].

mAbs are produced by recombinant technologies or eukaryotic organisms e.g., Chinese hamster ovary (CHO) cells. Eukaryotic cells can be engineered to express the protein in its glycosylated form. Producing full size antibodies in eukaryotic or cells is extremely labor intensive, costly, and problems arise with complex intellectual property (IP) issues associated with antibody composition and CDR, and the use of technical processes that are needed. Production and purification processes can be expensive, and coupled to research and then marketing costs can result in generally high drug costs, which ultimately limits patient access [7,107,108].

## 3. Potential Strategies to Overcome Challenges in Antibody-Based Therapies

### 3.1. Use of Excipients to Stabilize Formulations

Excipients have been used to increase the stability of a wide range of protein and peptide based formulations [109] by reducing protein dynamics and motion, increasing the conformational stability of mAbs especially at high concentrations and inhibiting interface-dependent aggregation [110,111,112]. Excipients usually inhibit aggregation and protects the protein by adsorbing to the air–liquid interface; for example, the use of surfactants (e.g., polysorbate 20 and 80), carbohydrates (e.g., cyclodextrin derivatives) and amino acids (e.g., arginine and histidine) can help prevent aggregation by this mechanism [103]. However, the use of polysorbate 80 can lead to micelle formation and hence, increase the chance of immunogenicity [103]. In one study, cyclodextrin was reported to stabilize commercially available antibody-based drugs in a hydrogel formulation [113]. Some of the generally recognized as safe (GRAS) excipients include pluronic F68, trehalose, glycine and amino acids such as arginine, glycine, glutamate and histidine, which are found in a number of commercial protein therapeutic products [110,114]. For example, Avastin^®^ (bevacizumab, 25 mg/mL) contains trehalose dehydrate, sodium phosphate and polysorbate 20. Excipients in subcutaneous Herceptin^®^ (trastuzumab, 600 mg) are rHuPH20, histidine hydrochloride, histidine, trehalose dehydrate, polysorbate 20, methionine and water for injection [115]. A number of studies have studied the use of excipients in the stabilization of mAbs [114,116,117]. The choice of excipients requires thorough screening and optimization to prevent aggregation [103]. One formulation excipient stabilizing a particular antibody might not be suitable for another antibody due to differences in their sequence [114,118].

### 3.2. Production of Protein Scaffolds

There is an increased focus on making more stable proteins so they can be formulated and used in long-acting forms. There are fundamental stability issues with mAbs, with the hinge region being the biggest problem. Several protein families with non-IgG architecture have been developed with novel binding motifs and are known as engineered protein scaffolds [12]. A scaffold is often defined as a single chain polypeptide framework that contains a highly structured core associated with variable portions of high conformational tolerance allowing insertions, deletions and other substitutions. Most scaffolds have been developed against validated targets including TNF-α, CD20, VEGF, CD19 and CD3 [7]. Scaffolds tend to be lower in molecular weight than mAbs and while they can share some structural features of an antibody, scaffolds have also been described that are unrelated to mAbs [7]. Protein scaffolds have been reported to have enhanced solubility and thermal stability and better tissue penetration [107]. It also offers a single polypeptide chain format and high bacterial expression for cheap production [119]. Many specialists have developed their own individual protein scaffold niches. Scaffolds can be either IgG- (e.g., nanobody, scFv, single domains) or non-IgG-like molecules (affibodies, anticalins, DARPins, dual-affinity retargeting molecules) and both categories have yielded smaller molecules that possess much of the epitope binding and specificity properties of mAbs. For example, non-IgG-like molecules possess higher stability, cysteine-free sequences and flexible pharmacokinetic properties [120].

Protein scaffolds do have some advantages over current protein therapeutics, however they also display some limitations too. As exogenous proteins, scaffold proteins are potentially immunogenic. Most protein therapeutics that have been developed during the last 30–40 years have been natural occurring proteins (e.g., cytokines, blood factors) or mAbs, which are very similar to endogenous IgGs. Minimizing immunogenicity is one major concern with the development of non-endogenous therapeutic proteins. For example, affibodies are derived from protein A, which is a bacterial protein. They generate an immune response after administration to human patients. Therefore, affibodies are targeted to be used as molecular imaging agents for cancer detection, instead of therapeutic agents [107]. Though most protein scaffolds are now being derived from naturally occurring human proteins, it still does not completely eliminate undesirable immune responses [107]. In addition, though there has been much investment to develop protein scaffolds, there is only one product (Kalbitor escallantide/DX-88), which has been successfully registered for clinical use [107].

### 3.3. mAb Formulations to Prolong the Duration of Action

**Figure 4:**
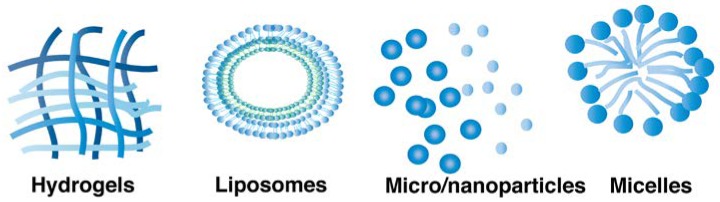
Common formulation strategies used to prolong protein release.

Formulation strategies have also been investigated to increase the duration of action of proteins, e.g., the preparation of controlled release systems (Figure 4). Here, more general approaches for the extension of protein action will be described that are also investigated for the development of longer acting mAb formulations.

#### 3.3.1. Microparticulate Associated Formulations

Microparticles have been examined for the long-term delivery of proteins including mAbs and peptides. The most common material used in microparticle formulations is the hydrolytically degradable co-polymer called poly(lactic-co-glycolic acid) (PLGA). Peptide loaded microparticles have been extensively described such as Exenatide, Sandostatin^®^, Vivitrol^®^, Risperdal^®^ Consta^®^, Zoladex and Lupron. As opposed to therapeutic peptides, the encapsulation of proteins into PLGA microspheres is quite challenging and not yet clinically approved due to the lack of protein stability. Loss of tertiary structure and biological activity can occur upon prolonged incubation with biological fluids under physiological conditions. PLGA polymers are hydrophobic, hence, different classes of protein molecules encapsulated by emulsion or phase separation are prone to surface adsorption, aggregation, denaturation, oxidation and cleavage leading to a loss in overall activity [121]. Often, there is a burst release with PLGA-based formulation and controlling the burst release can increase the duration of drug release. The burst release can be influenced by many factors such as the polymer composition [122], encapsulation process, and drying method [123].

Novel encapsulation technology could be used to overcome the above-mentioned limitations. A zero order release of bevacizumab from poly(caprolactone) (PCL) electrospun fibers was achieved over two months with the careful control of pH of loaded bevacizumab and encapsulation by electrohydrodynamic atomization (EHDA) [124]. The superior advantage of the EHDA is attributed to the process allowing encapsulation at ambient temperatures, which can preclude thermally-induced degradation. A number of other studies using microparticles for the delivery of antibodies have been reported. In one study, a potent intravitreal anti-VEGF formulation (dimeric molecule/dual dAb containing two different anti-VEGF domain antibodies (dAb) attached to a human IgG1 Fc region) using microparticles of PolyActive™ hydrogel co-polymer was reported to show a 6 month release [125]. In another study, infliximab was encapsulated into microspheres using either lyophilized particulate antibody or an aqueous solution of antibody, also known as thermally-induced phase separation (TIPS) technology. The ELISA experiment confirmed biological activity of infliximab against TNF-α. TIPS technology avoids the long-term exposure of proteins to oil/water interfaces and can prevent leaching and protein denaturation [126].

#### 3.3.2. Hydrogels and In Situ Forming Gels

A stated goal for hydrogel systems is to release the protein in its active form while maintaining the therapeutic concentration for at least 3 months [127]. Hydrogels are an alternative to particulate associated formulations and have been examined for the delivery of large molecular weight molecules [128], though no protein loaded hydrogel system is approved in the clinic yet. Hydrogels are polymeric materials that do not dissolve in water under physiological conditions and swell considerably in aqueous medium [129]. They are networks of polymer main chains covalently linked together, a process known as crosslinking, and sometimes the polymer crosslinks can be strong non-covalent interactions. The crosslinking of polymer chains prevents complete dissolution of the polymer. Hence, hydrogels made of hydrophilic polymers can imbibe water into their network structure and swell. The high water content properties of hydrogel makes them biocompatible and they are thus being examined in tissue regeneration applications. However, the high water content of hydrogels is a challenge for developing extended drug release formulations, although there are potential advantages of hydrogels compared to other drug delivery systems (DDS) [130].

Solidification of in situ gels can be triggered (without any crosslinking agents) with a change in solubility or temperature after injection. Phase inversion systems are a good example of in situ solidified implants. Often, the system composes of a protein drug suspended in polymer solution in a water-miscible organic solvent, thus forming a homogenous one-phase system. Upon injection, an organic solvent diffuses out thus causing polymer to precipitate, in which protein is effectively encapsulated in the in situ polymeric matrix. Slow degradable polymers similar to materials for micro/nanoparticles (e.g., PLGA, PLA and PCL) are commonly used. Polar organic solvent systems are preferable to dissolve polymers in the systems such as *N*-methyl pyrollidone (NMP), dimethyl sulfoxide (DMSO), ethanol, triacetin and glycofurol [131]. However, organic solvents in the system can de-stabilize protein structure and cause irritation upon injection [132]. The release profile of the gel systems depends on the gelling rate and porosity which influences by solvent system and polymers [133].

Thermo-responsive polymers have been described that are soluble in aqueous solution at 25 °C, which then undergo a sol-to-gel transition to form a collapsed, semi-solid form of the polymer at a different temperature, e.g., 33–37 °C. The lower critical solution temperature (LCST) is often used to indicate the temperature at which the sol-to-gel transition occurs. Often, thermo-responsive polymers are dissolved in aqueous systems, which can eliminate the problem associated with organic solvents [134]. *N*-isopropylacrylamide (NIPAAM) is a water-soluble polymer that can be prepared to have a LCST of ~37 °C. NIPAAM is soluble at ambient temperature but as temperature approaches the LCST, there is an increase of hydrophobic interactions that results in collapse of NIPAAM. Some of the protein mixed within the solubilized NIPAAM will inevitably be released from the NIPAAM polymer as it collapses. Once above the LCST, the collapsed NIPAAM decreases the diffusion of the remaining encapsulated protein [135]. Early preclinical studies with NIPAAM can be useful to develop a formulation strategy involving a therapeutic protein. Studies with thermally-responsive injectable NIPAAM polymers and NIPAAM crosslinked polymers have shown that an antibody and protein therapeutic can be encapsulated in the collapsed gel [129,136,137,138,139]. In one study, in vitro release samples of bevacizumab from a NIPAAM-loaded hydrogel showed binding to VEGF using surface plasmon resonance (SPR) from weeks 1–4 [129].

#### 3.3.3. Liposomes

Liposomes are bilayer vesicles consisting of phospholipids with both hydrophilic and hydrophobic compounds encapsulated in either the aqueous core of the vesicle or intercalated into the bilayer structure, respectively. Liposome formation is a result of the molecular alignment of phospholipids in water. Given the amphiphilic nature of the phospholipid structure, hydrophilic phosphate head groups are exposed to aqueous environment while the hydrocarbon chains interact with each other thus forming a lipid film. The lipid sheet becomes enclosed vesicles upon adding water and stirring [140]. Liposomes can contain one bilayer (unilamellar) or multiple bilayers (multi-lamellar), with the former varying in size from approximately 20–100 nm vesicles (small unilamellar vesicles, SUVs) to larger vesicles at around 100–1000 nm (large unilamellar vesicles, LUVs) [140]. Dry lipid hydration, freeze-thawing extrusion, reverse evaporation and double emulsification are some of the techniques used to prepare liposomes [140]. In brief, all involve the hydration of a lipid film, subsequent mechanical dispersion to form liposomes, and solvent removal. Vigorous shaking is commonly used to disperse liposomes, however, it typically generates polydispersed MLVs [141]. Therefore, the size of liposomes can be controlled and reduced by extrusion through a small orifice to obtain mono-dispersed SUV liposomes [142]. However, many physical stresses (e.g., the use of heat, organic solvents and agitation) are involved during the preparation of liposomes, and can compromise the stability of the encapsulated proteins [143].

Many researchers have reported the increased bioavailability of biotherapeutics incorporated into liposomes. Unlike polymeric particles, conventional liposomal systems are less likely to prolong the release of the encapsulated proteins. The effect of pH on bilayer destabilization can account for the breakdown of liposomes and therefore, releasing encapsulated agents [144]. Processes such as protonation of the phospholipid head group and acid-catalyzed hydrolysis of the bilayer can lead to the in vivo breakdown of the bilayer. Modification of the lipid bilayer can alter drug kinetic profile, e.g., the type of phospholipids or the incorporation of cholesterol into the layers [145]. Functionalizing the liposomal surface with PEGylation [146] can prevent liposome aggregation and can enhance stability by decreasing the interactions of protein with biological fluids [147].

### 3.4. Protein Modification to Increase Duration of Action

**Figure 5:**
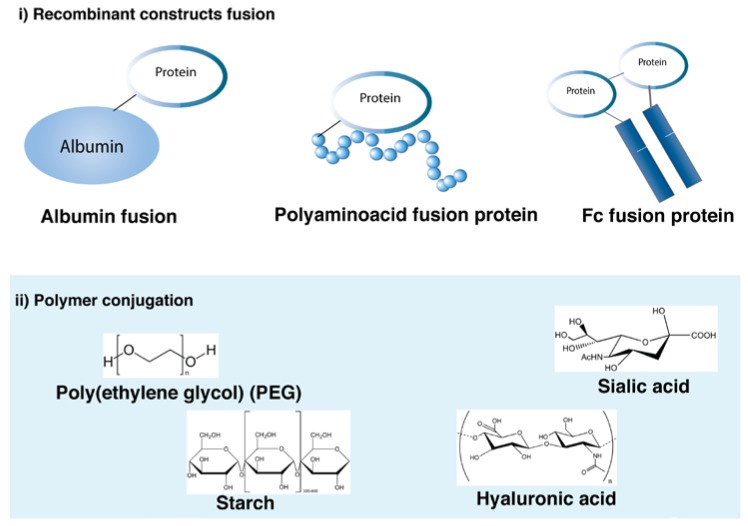
Methods to increase the duration of action of proteins.

Recombinant and chemical modifications to extend the half-life of proteins are summarized in Figure 5. Most of the strategies have been used for the extension of protein action and can be used for long-acting mAb formulations. Human serum albumin (HSA), Fc fusion and PEGylation are some of the strategies that have either entered or passed clinical trials and are discussed in more detail in this section.

#### 3.4.1. Albumin–Protein Fusions

Albumin is the most abundant protein in the blood and it is a multi-binding transporter protein produced by the liver. It has various binding capacities toward different insoluble and hydrophobic endogenous and exogenous ligands. Albumin has three domains (DI, DII, DIII) each having two sub domains (A and B) connected by a flexible loop. The domains possess biological activity and have seven binding sites for fatty acids. Albumin has a half-life of 19–22 days in humans, which is made possible by FcRn-mediated recycling analogous to mAbs [148]. The FcRn-binding site is in DIII of albumin. Proteins can be fused either to the N- or C-terminus of albumin in an effort to exploit the long half-life of albumin. A small fragment (22 kDa) of albumin containing the FcRn binding domain is capable of extending the half-life of a protein therapeutic [148].

Although an albumin–peptide fusion has been registered for clinical use, no albumin–protein fusion product has yet been successful in clinical trials. The albumin–peptide fusion product, Albiglutide (Tanzeum^®^), is a recombinant fusion protein with two tandem copies of modified human GLP fused to albumin that was approved to improve glycemic control with adults with type 2 diabetes mellitus. Albiglutide has a molecular weight of about 73 kDa and acts as a GLP agonist. The albumin has further been engineered to be resistant to DPP-4 mediated proteolysis. Albiglutide improves the half-life of active GLP-1 from 1–2 min for native GLP-1 to 4–7 days allowing once weekly dosing [149]. GlaxoSmithKline (GSK) and Abylnx have developed single domain antibodies such as nanobodies that bind to albumin [150]. For example, GSK2374697 is a novel albumin-binding domain antibody that has been reported to have high affinity to HSA resulting in a long-duration GLP-1 receptor agonist [151].

A number of studies using albumin fusions for antibody conjugation have been reported in the literature. One study reported stability of trastuzumab conjugates using HSA domain I in human plasma [152]. Another study discussed the preparation of three recombinant antibody formats using two different scFv molecules, bispecific single chain diabodies and tandem scFv, respectively to HSA targeted to tumor CEA. The study demonstrated increased circulation time and high stability up to 24 days at 37 °C [153]. In another study, the preparation of a scFv and HSA conjugate was discussed as a potential immunosuppressive therapy of myasthenia gravis. The mean inhibition rate of binding to the acetylcholine receptor was 31.4% for 3 days [154]. Another study investigated the different binding of HSA to FcRn across different species. A scFv–albumin fusion conjugate was found to only slightly reduce in human FcRn binding, whereas the binding was drastically reduced in rodent FcRn [155].

#### 3.4.2. Fc Fusion Proteins

Fc fusion proteins are therapeutic proteins or peptides that have been recombinantly fused to the Fc found in mAbs. Fc fusion proteins can endow peptides or proteins with IgG-like pharmacokinetic properties and a longer serum half-life by FcRn recycling mechanism. Virtually all types of Fc fusion proteins have been designed for the purpose of half-life extension. Etanercept (Enbrel^®^, Amgen/Pfizer) is by far the most successful from the eight first generation products. It was the first approved Fc fusion therapeutic in 1998 and was the highest selling protein therapeutic in 2009 with $6.6 billion sales worldwide [108]. Other first-generation Fc-fusion proteins include alefacept (Amevive^®^), abatacept (Orencia^®^), rilonacept (Arcalyst^®^), romiplostim (Nplate^®^), belatacept (Nulojix^®^), aflibercept (Eylea^®^) and ziv-aflibercept (Zaltrap^®^). New Fc fusion proteins continue to be developed and now Fc fusion proteins that are considered as biobetters have also been developed including denileukin diftitox (Ontak^®^), corifollitropin- α (Elonva^®^), eftrenonacog-α (Alprolix^®^), albiglutide (Tanzeum^®^), efraloctocog-α (Eloctate^®^)and dulaglutide (Trulicity^®^) [149]. Most of the Fc-fusion proteins target receptor-ligand interactions either as antagonists to block receptor binding or as agonists to stimulate receptor functions [156].

Aflibercept (Eylea^®^/VEGF Trap-Eye) has been approved for intraocular use to treat subfoveal choroidal neovascularization due to AMD. Aflibercept incorporates the second binding domain of the VEGFR-1 receptor and the third domain of the VEGFR-2 receptor and binds to VEGF-A, VEGF-B and placental growth factor 1 and 2 [157]. In humans, the half-life of aflibercept increased with an increase in dose after intravenous administration of aflibercept, ranging from 1.7 days (0.3 mg/kg cohort) to 7.4 days (5.0 mg/kg cohort) [158]. However, no human pharmacokinetic data of aflibercept is available after intravitreal administration [159]. An aflibercept (2.0 mg, 50 μL) half-life of 1.5 and 2.2 days was found in the aqueous humor of vitrectomized and non-vitrectomized macaque eyes respectively [160]. In one study, patients were randomized and received different intravitreal doses of aflibercept i.e., 0.5 mg monthly, 2.0 mg monthly and 2.0 mg every 2 months after 3 initial monthly doses. The comparator was 0.5 mg ranibizumab, which was the standard clinical dose used at the time. Intravitreal injection of aflibercept dosed monthly or every 2 months after 3 initial monthly doses produced similar efficacy and safety outcomes as monthly ranibizumab in the treatment of AMD. It was determined that the 2.0 mg dose of aflibercept could be administered every 2 months to reduce the risk of monthly intravitreal injections [161]. It should be noted that the 2.0 mg dose of aflibercept is greater on a molar basis than that of the 0.5 mg clinical dose of ranibizumab. Ziv-aflibercept (Zaltrap^®^) was approved in 2012 for the treatment of metastatic colorectal cancer and for patients whose immune systems are not functioning normally [128]. The difference between VEGF Trap-Eye and Zaltrap is their osmolarity. The osmolarity is 250 to 260 mOsm and 815 to 820 mOsm for VEGF Trap-Eye and Zaltrap respectively [162]. Hyperosmotic solutions lead to retinal detachment [163]. However, in a preclinical investigation of intravitreal Zaltrap was reported to be safe in rabbit retina [164].

#### 3.4.3. Protein PEGylation

PEGylation is the covalent conjugation of PEG to a protein. The PEG–protein conjugate is considered to be new chemical entity (NCE) usually with improved properties. PEG can prolong the half-life of biotherapeutics because each repeat unit in the polymer can form hydrogen bonds with three molecules of water, thus increasing the hydrodynamic volume [165]. An increase in protein molecular weight can reduce the impact of processes such as glomerular excretion and immunogenicity [166]. Linear PEG is derived from ethylene oxide repeat units (HO-(CH_2_CH_2_O)n-H). PEG is typically activated at one terminus for protein conjugation with a non-reactive methyl group at the other terminus. The metabolism of PEG involves the oxidation of PEG hydroxy to carboxylic acid, which generates minor amounts of oxalic acid. P450s play a role in the oxidation of PEG with evidence that the phase 1 metabolism also involves enzymatic metabolism by alcohol dehydrogenase and sulfotransferase [167].

The first PEGylated product to be approved was pegademase (Adagen^®^) for the treatment of severe combined immunodeficiency disease (SCID). All FDA approved PEG products are PEG conjugated proteins except Peginesatide (Omontys^®^) and Pegaptanib (Macugen^®^). Peginesatide is a PEGylated peptide developed as an alternative to erythropoietin to avoid PRCA, which is an immune reaction against erythropoietin. Unfortunately, after registration, it was found that there were cardiovascular toxicities associated with this product resulting in its withdrawal. The withdrawal was due to the activity of the peptide and not to any toxicity associated with PEG. Pegaptanib (Macugen^®^) is a PEGylated RNA aptamer that binds to VEGF approved in 2004 for the treatment of wet AMD [168]. The approval of pegaptanib was a milestone in drug development as it was the first aptamer to be successfully developed as a therapeutic agent for humans. It is also the first anti-angiogenic therapy indicated for the treatment of neovascular AMD [168]. Receptor activation in the eye is prevented when pegaptanib binds to VEGF_165_, which averts choroidal neovascularization (CNV) with the reduction of vascular permeability. As a result, macular edema associated with AMD can be avoided [18]. Pegaptanib consists of two 20 kDa monomethoxy PEG units covalently attached to the two amino groups of a lysine linker that is activated at the lysine carboxyl group for conjugation to the aptamer [169]. As a result of pegaptanib binding to a specific VEGF isoform, pegaptanib has limited efficacy compared to ranibizumab and bevacizumab. Due to its limited targeting (and efficacy), the adverse effects of pegaptanib are reduced compared to other ranibizumab and bevacizumab [170]. An intravitreal dose of pegaptanib (0.3 mg) showed a half-life of 7–8 days [171].

Certolizumab pegol (Cimzia^®^) is an *Escherichia. coli* derived Fab’ that binds to TNF-α that is PEGylated with a similar branched 40 kDa PEG reagent used in pegaptanib. The PEG reagent used for certolizumab compared to that used for pegaptanib is selective for the free hinge cysteine thiol on the Fab’ [148]. The carboxyl group on the branched 40 kDa PEG reagent has been derivatized with a thiol-selective maleimide moiety whereas the carboxyl group on branched 40 kDa PEG reagent used for pegaptanib was derivatized as a *N*-hydroxysuccinimide ester designed to undergo reaction with amines. Cimzia^®^ is approved in most of the regulated markets for the treatment of rheumatoid arthritis and Crohn’s disease. There are several clinically used antibody-based TNF-α inhibitors including infliximab (Remicade^®^), adalimumab (Humira^®^), etanercept (Enbrel^®^) and golimumab (Simponi^®^). There are also antibodies for other targets that have been developed to treat rheumatoid arthritis include abatacept (Orencia^®^), rituximab (Rituxan^®^) and toclizumab (Actemra^®^). Additionally, anakinra (Kineret^®^), which is a recombinant protein that is an interleukin 1 receptor antagonist, is also sometimes used to treat rheumatoid arthritis. Certolizumab pegol has a similar safety profile to other TNF-α inhibitors and has a favorable low level of injection site pain. It differs from other TNF-α inhibitors as it lacks an Fc region, which reduces the Fc-mediated effects such as CDC or ADCC [172]. The covalent conjugation of PEG to Fab’ results in a *t*_1/2_ of ~2 weeks with a 2 to 4 weeks dosing schedule [169].

There has been concern about potential PEG toxicity and immunogenicity. Formation of vacuoles in the kidney has been reported with high doses of PEG in animals [121,173]. These vacuoles disappear when the dosing of PEG is stopped. In humans there does not appear to be any clinical toxicity of PEG. It is clear that high doses of any water-soluble polymer that is essentially non-hydrolytically degradable will accumulate in humans, but doses of PEG-proteins tend to be low (generally less than 1 mg/kg) except for certolizumab pegol, which is 200 mg, and no PEG toxicity has been reported. The only toxicities observed clinically with PEG-protein conjugates are those associated with the protein [167].

In terms of immunogenicity, since PEG is widely used in consumer products, there is concern that many people have developed secondary antibodies to PEG [174]. It is thought the main epitope in PEG–protein conjugates is the terminal methyl group on PEG [175]. Since PEG is a highly flexible molecule, the affinity of secondary antibodies is low. Secondary antibody clearance is only seen with non-human enzymes that have many molecules of conjugated PEG to each enzyme molecule [176]. Such enzymes (e.g., uricase, asparagenase) have small molecule substrates, so hyper PEGylation still yields PEG–enzyme conjugates that are clinically beneficial. These non-human enzymes could not be used clinically beyond a small number of doses without having been PEGylated. The vast majority of PEGylated proteins only have one molecule of PEG conjugated to the protein, so immunogenicity is much less observed.

PEG sterically shields the conjugated protein. While this reduces protein degradation by proteolysis to help with increasing the circulation time of the protein, there is often a reduction of the in vitro biological activity of the protein after PEGylation [177]. Many therapeutic proteins are highly potent, so even with an in vitro activity loss of 95%, they remain clinically beneficial over the unmodified protein. PEGylation does not change protein function, only the rate of association to its target. Once the PEGylated protein is bound to its target, it will dissociate at the same rate as the unmodified protein. The reduced biological activity is caused by the reduce rate of association to the target. PEG steric shielding is a key cause for reducing the rate of association of the protein to its target. Many PEGylation reagents undergo conjugation non-specifically at different residues resulting in positional isomers that give PEG-protein conjugates with different biological activities [167]. Also, many PEGylation reagents must be used at an excess stoichiometry, which requires tedious and expensive purification processes. Efficient site-specific and site-selective PEGylation strategies have been developed in recent years in efforts to improve the biological activity and purification processes of PEG–protein conjugates [169,178].

## 4. Conclusions

Monoclonal antibodies continue to be one of the fastest growing categories of protein therapeutics. The development of aggregation and unwanted immunogenicity can lead to various manufacturing and clinical challenges. The stability of mAbs can be enhanced with the use of excipients (e.g., surfactants and amino acids) or by the production of more stable constructs (e.g., protein scaffolds and bispecific molecules). A number of technologies (e.g., Fc fusions and PEGylation) have also been used to improve the pharmacokinetic and stability profiles of mAbs.

## References

[1] Keizer R.J., Huitema A.D.R., Schellens J.H.M., Beijnen J.H. (2010). Clinical pharmacokinetics of therapeutic monoclonal antibodies. Clin. Pharmacokinet..

[2] Liu J.K.H. (2014). The history of monoclonal antibody development—Progress, remaining challenges and future innovations. Ann. Med. Surg..

[3] Smith S.L. (1996). Ten years of Orthoclone OKT3 (muromonab-CD3): A review. J. Transpl. Coord..

[4] Beck A., Wagner-Rousset E., Bussat M.C., Lokteff M., Klinguer-Hamour C., Haeuw J.F., Goetsch L., Wurch T., Dorsselaer A.V., Corvaïa N. (2008). Trends in glycosylation, glycoanalysis and glycoengineering of therapeutic antibodies and Fc-fusion proteins. Curr. Pharm. Biotechnol..

[5] Mahmuda A., Bande F., Jameel K., Abdulhaleem N., Majid R.A., Hamat R.A., Abdullah W.O., Unyah Z. (2017). Monoclonal antibodies: A review of therapeutic applications and future prospects. Trop. J. Pharm. Res..

[6] Rodrigues E.B., Farah M.E., Maia M., Penha F.M., Regatieri C., Melo G.B., Pinheiro M.M., Zanetti C.R. (2009). Therapeutic monoclonal antibodies in ophthalmology. Prog. Retin. Eye Res..

[7] Wurch T., Pierré A., Depil S. (2012). Novel protein scaffolds as emerging therapeutic proteins: From discovery to clinical proof-of-concept. Trends Biotechnol..

[8] Ecker D.M., Jones S.D., Levine H.L. (2015). The therapeutic monoclonal antibody market. MAbs.

[9] Attaelmannan M., Levinson S.S. (2000). Understanding and identifying monoclonal gammopathies. Clin. Chem..

[10] Vidarsson G., Dekkers G., Rispens T. (2014). IgG subclasses and allotypes: From structure to effector functions. Front. Immunol..

[11] Raju T.S., Scallon B.J. (2006). Glycosylation in the Fc domain of IgG increases resistance to proteolytic cleavage by papain. Biochem. Biophys. Res. Commun..

[12] Gebauer M., Skerra A. (2009). Engineered protein scaffolds as next-generation antibody therapeutics. Curr. Opin. Chem. Biol..

[13] Dirks N.L., Meibohm B. (2010). Population pharmacokinetics of therapeutic monoclonal antibodies. Clin. Pharmacokinet..

[14] Thakur A., Lum L.G. (2016). “NextGen” Biologics: Bispecific Antibodies and Emerging Clinical Results. Expert Opin. Biol. Ther..

[15] Porter R.R. (1958). Separation and isolation of fractions of rabbit gamma-globulin containing the antibody and antigenic combining sites. Nature.

[16] Zhao Y., Gutshall L., Jiang H., Baker A., Beil E., Obmolova G., Carton J., Taudte S., Amegadzie B. (2009). Two routes for production and purification of Fab fragments in biopharmaceutical discovery research: Papain digestion of mAb and transient expression in mammalian cells. Protein Expr. Purif..

[17] Herrington-Symes A.P., Farys M., Khalili H., Brocchini S. (2013). Antibody fragments: Prolonging circulation half-life special issue-antibody research. Adv. Biosci. Biotechnol..

[18] Kourlas H., Schiller D.S. (2006). Pegaptanib sodium for the treatment of neovascular age-related macular degeneration: A review. Clin. Ther..

[19] Rosenfeld P.J., Heier J.S., Hantsbarger G., Shams N. (2006). Tolerability and Efficacy of Multiple Escalating Doses of Ranibizumab (Lucentis) for Neovascular Age-Related Macular Degeneration. Ophthalmology.

[20] Rosenfeld P.J., Schwartz S.D., Blumenkranz M.S., Miller J., Haller J., Reimann J., Greene W., Shams N. (2005). Maximum tolerated dose of a humanized anti-vascular endothelial growth factor antibody fragment for treating neovascular age-related macular degeneration. Ophthalmology.

[21] Heier J.S., Antoszyk A.N., Pavan P.R., Leff S.R., Rosenfeld P.J., Ciulla T.A., Dreyer R.F., Gentile R.C., Sy J.P., Hantsbarger G. (2006). Ranibizumab for Treatment of Neovascular Age-Related Macular Degeneration. A Phase I/II Multicenter, Controlled, Multidose Study. Ophthalmology.

[22] Nelson A.L. (2010). Antibody fragments: Hope and hype. MAbs.

[23] Stewart M.W. (2014). Pharmacokinetics, pharmacodynamics and pre-clinical characteristics of ophthalmic drugs that bind VEGF. Expert Rev. Clin. Pharmacol..

[24] Wright A., Morrison S.L. (1997). Effect of glycosylation on antibody function: Implications for genetic engineering. Trends Biotechnol..

[25] Desjarlais J.R., Lazar G.A. (2011). Modulation of antibody effector function. Exp. Cell Res..

[26] Goswami S., Wang W., Arakawa T., Ohtake S. (2013). Developments and Challenges for mAb-Based Therapeutics. Antibodies.

[27] Clynes R.A., Towers T.L., Presta L.G., Ravetch J.V. (2000). Inhibitory Fc receptors modulate in vivo cytoxicity against tumor targets. Nat. Med..

[28] Brennan F.R., Morton L.D., Spindeldreher S., Kiessling A., Allenspach R., Hey A., Müller P., Frings W., Sims J. (2010). Safety and immunotoxicity assessment of immunomodulatory monoclonal antibodies. MAbs.

[29] Jiang X.R., Song A., Bergelson S., Arroll T., Parekh B., May K., Chung S., Strouse R., Mire-Sluis A., Schenerman M. (2011). Advances in the assessment and control of the effector functions of therapeutic antibodies. Nat. Rev. Drug Discov..

[30] Roopenian D.C., Akilesh S. (2007). FcRn: The neonatal Fc receptor comes of age. Nat. Rev. Immunol..

[31] Anderson C.L., Chaudhury C., Kim J., Bronson C.L., Wani M.A., Mohanty S. (2006). Perspective—FcRn transports albumin: Relevance to immunology and medicine. Trends Immunol..

[32] Andersen J.T., Pehrson R., Tolmachev V., Daba M.B., Abrahmsén L., Ekblad C. (2011). Extending half-life by indirect targeting of the neonatal Fc receptor (FcRn) using a minimal albumin binding domain. J. Biol. Chem..

[33] Kim H., Robinson S.B., Csaky K.G. (2009). FcRn receptor-mediated pharmacokinetics of therapeutic IgG in the eye. Mol. Vis..

[34] Sockolosky J.T., Szoka F.C. (2015). The neonatal Fc receptor, FcRn, as a target for drug delivery and therapy. Adv. Drug Deliv. Rev..

[35] Kobayashi K., Qiao S.W., Yoshida M., Baker K., Lencer W.I., Blumberg R.S. (2009). An FcRn-Dependent Role for Anti-flagellin Immunoglobulin G in Pathogenesis of Colitis in Mice. Gastroenterology.

[36] Zhang Z., Chen X., Hernandez L.D., Lipari P., Flattery A., Chen S.-C., Kramer S., Polishook J.D., Racine F., Cape H. (2015). Toxin-mediated paracellular transport of antitoxin antibodies facilitates protection against Clostridium difficile infection. Infect. Immun..

[37] Shah U., Dickinson B.L., Blumberg R.S., Simister N.E., Lencer W.I., Walker W.A. (2003). Distribution of the IgG Fc receptor, FcRn, in the human fetal intestine. Pediatr. Res..

[38] Pang G., Wang Y., Xie J., Chen Q., Hu Z. (2015). Human FcRn can mediate the transport across intestinal mucosal barrier and prolong the half-life of rabbit IgG In Vivo. Braz. Arch. Biol. Technol..

[39] Hornby P.J., Cooper P.R., Kliwinski C., Ragwan E., Mabus J.R., Harman B., Thompson S., Kauffman A.L., Yan Z., Tam S.H. (2014). Human and non-human primate intestinal FcRn expression and immunoglobulin G transcytosis. Pharm. Res..

[40] Vogelzang A., Lozza L., Reece S.T., Perdomo C., Zedler U., Hahnke K., Oberbeck-Mueller D., Dorhoi A., Kaufmann S.H.E. (2016). Neonatal Fc Receptor Regulation of Lung Immunoglobulin and CD103^+^ Dendritic Cells Confers Transient Susceptibility to Tuberculosis. Infect. Immun..

[41] Bitonti A.J., Dumont J.A., Low S.C., Peters R.T., Kropp K.E., Palombella V.J., Stattel J.M., Lu Y., Tan C.A., Song J.J. (2004). Pulmonary delivery of an erythropoietin Fc fusion protein in non-human primates through an immunoglobulin transport pathway. Proc. Natl. Acad. Sci. USA.

[42] Shatz W., Hass P.E., Mathieu M., Kim H.S., Leach K., Zhou M., Crawford Y., Shen A., Wang K., Chang D.P. (2016). Contribution of Antibody Hydrodynamic Size to Vitreal Clearance Revealed through Rabbit Studies Using a Species-Matched Fab. Mol. Pharm..

[43] Gadkar K., Pastuskovas C.V., Le Couter J.E., Elliott J.M., Zhang J., Lee C.V., Sanowar S., Fuh G., Kim H.S., Lombana T.N. (2015). Design and pharmacokinetic characterization of novel antibody formats for ocular therapeutics. Investig. Ophthalmol. Vis. Sci..

[44] Birch J.R., Racher A.J. (2006). Antibody production. Adv. Drug Deliv. Rev..

[45] Steinmeyer D.E., McCormick E.L. (2008). The art of antibody process development. Drug Discov. Today.

[46] Roque A.C.A., Lowe C.R., Taipa M.Â. (2004). Antibodies and genetically engineered related molecules: Production and purification. Biotechnol. Prog..

[47] Gronemeyer P., Ditz R., Strube J. (2014). Trends in Upstream and Downstream Process Development for Antibody Manufacturing. Bioengineering.

[48] Epstein N., Epstein M. (1986). The hybridoma technology: I. Production of monoclonal antibodies. Adv. Biotechnol. Processes.

[49] Little M., Kipriyanov S.M., Le Gall F., Moldenhauer G. (2000). Of mice and men: Hybridoma and recombinant antibodies. Immunol. Today.

[50] Tomita M., Tsumoto K. (2011). Hybridoma technologies for antibody production. Immunotherapy.

[51] Nelson A.L., Dhimolea E., Reichert J.M. (2010). Development trends for human monoclonal antibody therapeutics. Nat. Rev. Drug Discov..

[52] Geyer C.R., McCafferty J., Dübel S., Bradbury A.R.M., Sidhu S.S. (2012). Recombinant Antibodies and In Vitro Selection Technologies. Antibody Methods and Protocols.

[53] Kuhn P., Fühner V., Unkauf T., Moreira G.M.S.G., Frenzel A., Miethe S., Hust M. (2016). Recombinant antibodies for diagnostics and therapy against pathogens and toxins generated by phage display. Proteom. Clin. Appl..

[54] Frenzel A., Schirrmann T., Hust M. (2016). Phage display-derived human antibodies in clinical development and therapy. MAbs.

[55] Bradbury A.R.M., Sidhu S., Dübel S., McCafferty J. (2011). Beyond natural antibodies: The power of in vitro display technologies. Nat. Biotechnol..

[56] McCafferty J., Glover D.R. (2000). Engineering therapeutic proteins. Curr. Opin. Struct. Biol..

[57] Constantinou A., Epenetos A.A., Hreczuk-Hirst D., Jain S., Wright M., Chester K.A., Deonarain M.P. (2009). Site-specific polysialylation of an antitumor single-chain Fv fragment. Bioconjug. Chem..

[58] Kontermann R.E., Brinkmann U. (2015). Bispecific antibodies. Drug Discov. Today.

[59] Fan G., Wang Z., Hao M., Li J. (2015). Bispecific antibodies and their applications. J. Hematol. Oncol..

[60] Neuberger M. (1996). Generating high-avidity human Mabs in mice. Nat. Biotechnol..

[61] Almeida A.J., Souto E. (2007). Solid lipid nanoparticles as a drug delivery system for peptides and proteins. Adv. Drug Deliv. Rev..

[62] Chakravarthy U., Harding S.P., Rogers C.A., Downes S.M., Lotery A.J., Wordsworth S., Reeves B.C. (2012). Ranibizumab versus bevacizumab to treat neovascular age-related macular degeneration: One-year findings from the IVAN randomized trial. Ophthalmology.

[63] Martin D.F., Maguire M.G., Fine S.L., Ying G.S., Jaffe G.J., Grunwald J.E., Toth C., Redford M., Ferris F.L. (2012). Ranibizumab and bevacizumab for treatment of neovascular age-related macular degeneration: Two-year results. Ophthalmology.

[64] Moisseiev E., Waisbourd M., Ben-Artsi E., Levinger E., Barak A., Daniels T., Csaky K., Loewenstein A., Barequet I.S. (2014). Pharmacokinetics of bevacizumab after topical and intravitreal administration in human eyes. Graefe Arch. Clin. Exp. Ophthalmol..

[65] Meyer C.H., Krohne T.U., Holz F.G. (2011). Intraocular pharmacokinetics after a single intravitreal injection of 1.5 mg versus 3.0 mg of bevacizumab in humans. Retina.

[66] Zhu Q., Ziemssen F., Henke-Fahle S., Tatar O., Szurman P., Aisenbrey S., Schneiderhan-Marra N., Xu X., Grisanti S. (2008). Vitreous levels of bevacizumab and vascular endothelial growth factor-A in patients with choroidal neovascularization. Ophthalmology.

[67] Beer P.M., Wong S.J., Hammad A.M., Falk N.S., O’Malley M.R., Khan S. (2006). Vitreous levels of unbound bevacizumab and unbound vascular endothelial growth factor in two patients. Retina.

[68] Jager R.D., Aiello L.P., Patel S.C., Cunningham E.T. (2004). Risks of intravitreous injection: A comprehensive review. Retina.

[69] Dickmann L. (2016). Ocular therapeutics: Drug delivery and pharmacology. Mol. Pharm..

[70] Samaranayake H., Wirth T., Schenkwein D., Räty J.K., Ylä-Herttuala S. (2009). Challenges in monoclonal antibody-based therapies. Ann. Med..

[71] Shi S. (2014). Biologics: An update and challenge of their pharmacokinetics. Curr. Drug Metab..

[72] Takakura Y., Mahato R.I., Nishikawa M., Hashida M. (1996). Control of pharmacokinetic profiles of drug-macromolecule conjugates. Adv. Drug Deliv. Rev..

[73] Supersaxo A., Hein W.R., Steffen H. (1990). Effect of molecular weight on the lymphatic absorption of water-soluble compounds following subcutaneous administration. Pharm. Res..

[74] Richter W.F., Bhansali S.G., Morris M.E. (2012). Mechanistic Determinants of Biotherapeutics Absorption Following SC Administration. AAPS J..

[75] Baumann A. (2006). Early development of therapeutic biologics—Pharmacokinetics. Curr. Drug Metab..

[76] Barar J., Javadzadeh A.R., Omidi Y. (2008). Ocular novel drug delivery: Impacts of membranes and barriers. Expert Opin. Drug Deliv..

[77] Shpilberg O., Jackisch C. (2013). Subcutaneous administration of rituximab (MabThera) and trastuzumab (Herceptin) using hyaluronidase. Br. J. Cancer.

[78] Leveque D. (2014). Subcutaneous administration of anticancer agents. Anticancer Res..

[79] Frost G.I. (2007). Recombinant human hyaluronidase (rHuPH20): An enabling platform for subcutaneous drug and fluid administration. Expert Opin. Drug Deliv..

[80] Pereira De Sousa I., Bernkop-Schnürch A. (2014). Pre-systemic metabolism of orally administered drugs and strategies to overcome it. J. Control. Release.

[81] Shankar G., Shores E., Wagner C., Mire-Sluis A. (2006). Scientific and regulatory considerations on the immunogenicity of biologics. Trends Biotechnol..

[82] Harding F.A., Stickler M.M., Razo J., DuBridge R.B. (2010). The immunogenicity of humanized and fully human antibodies. MAbs.

[83] Awwad S., Lockwood A., Brocchini S., Khaw P.T. (2015). The PK-Eye: A Novel In Vitro Ocular Flow Model for Use in Preclinical Drug Development. J. Pharm. Sci..

[84] Brinch K.S., Frimodt-Møller N., Høiby N., Kristensen H.-H. (2009). Influence of antidrug antibodies on plectasin efficacy and pharmacokinetics. Antimicrob. Agents Chemother..

[85] Brinks V., Jiskoot W., Schellekens H. (2011). Immunogenicity of therapeutic proteins: The use of animal models. Pharm. Res..

[86] Van Beers M.M.C., Sauerborn M., Gilli F., Brinks V., Schellekens H., Jiskoot W. (2010). Aggregated recombinant human interferon Beta induces antibodies but no memory in immune-tolerant transgenic mice. Pharm. Res..

[87] Tabrizi M.A., Tseng C.M., Roskos L.K. (2006). Elimination mechanisms of therapeutic monoclonal antibodies. Drug Discov. Today.

[88] Wang B., Lau Y.Y., Liang M., Vainshtein I., Zusmanovich M., Lu H., Magrini F., Sleeman M., Roskos L. (2012). Mechanistic modeling of antigen sink effect for mavrilimumab following intravenous administration in patients with rheumatoid arthritis. J. Clin. Pharmacol..

[89] McKeage K., Perry C.M. (2002). Trastuzumab: A review of its use in the treatment of metastatic breast cancer overexpressing HER2. Drugs.

[90] Baert F., Noman M., Vermeire S., Van Assche G., D’Haens G., Carbonez A., Rutgeerts P. (2003). Influence of immunogenicity on the long-term efficacy of infliximab in Crohn’s disease. N. Engl. J. Med..

[91] Roberts C.J. (2014). Therapeutic protein aggregation: Mechanisms, design, and control. Trends Biotechnol..

[92] Mahler H.C., Friess W., Grauschopf U., Kiese S. (2009). Protein aggregation: Pathways, induction factors and analysis. J. Pharm. Sci..

[93] Philo J.S., Arakawa T. (2009). Mechanisms of protein aggregation. Curr. Pharm. Biotechnol..

[94] Neergaard M.S., Nielsen A.D., Parshad H., De Weert M.V. (2014). Stability of Monoclonal Antibodies at High-Concentration: Head-to-Head Comparison of the IgG1 and IgG4 Subclass. J. Pharm. Sci..

[95] Frokjaer S., Otzen D.E. (2005). Protein drug stability: A formulation challenge. Nat. Rev. Drug Discov..

[96] Manning M.C., Chou D.K., Murphy B.M., Payne R.W., Katayama D.S. (2010). Stability of protein pharmaceuticals: An update. Pharm. Res..

[97] Chavez B.K., Agarabi C.D., Read E.K., Boyne M.T., Khan M.A., Brorson K.A. (2016). Improved Stability of a Model IgG3 by DoE-Based Evaluation of Buffer Formulations. Biomed. Res. Int..

[98] Pikal-Cleland K.A., Cleland J.L., Anchordoquy T.J., Carpenter J.F. (2002). Effect of glycine on pH changes and protein stability during freeze-thawing in phosphate buffer systems. J. Pharm. Sci..

[99] Roy S., Henderson I., Nayar R., Randolph T.W., Carpenter J.F. (2008). Effect of pH on stability of recombinant botulinum serotype A vaccine in aqueous solution and during storage of freeze-dried formulations. J. Pharm. Sci..

[100] Angkawinitwong U., Sharma G., Brocchini S. (2015). Solid-state protein formulation. Ther. Deliv..

[101] Farrell R.A., Marta M., Gaeguta A.J., Souslova V., Giovannoni G., Creeke P.I. (2012). Development of resistance to biologic therapies with reference to IFN-β. Rheumatology.

[102] McKoy J.M., Stonecash R.E., Cournoyer D., Rossert J., Nissenson A.R., Raisch D.W., Casadevall N., Bennett C.L. (2008). Epoetin-associated pure red cell aplasia: Past, present, and future considerations. Transfusion.

[103] Ratanji K.D., Derrick J.P., Dearman R.J., Kimber I. (2014). Immunogenicity of therapeutic proteins: Influence of aggregation. J. Immunotoxicol..

[104] Arriola-Villalobos P., Martinez-de-la-Casa J.M., Diaz-Valle D., Morales-Fernandez L., Fernandez-Perez C., Garcia-Feijoo J. (2016). Glaukos iStent inject^®^ Trabecular Micro-Bypass Implantation Associated with Cataract Surgery in Patients with Coexisting Cataract and Open-Angle Glaucoma or Ocular Hypertension: A Long-Term Study. J. Ophthalmol..

[105] Kossovsky N., Heggers J.P., Robson M.C. (1987). Experimental demonstration of the immunogenicity of silicone–Protein complexes. J. Biomed. Mater. Res..

[106] Thirumangalathu R., Krishnan S., Ricci M.S., Brems D.N., Randolph T.W., Carpenter J.F. (2009). Silicone oil- and agitation-induced aggregation of a monoclonal antibody in aqueous solution. J. Pharm. Sci..

[107] Mintz C.S., Crea R. (2013). Protein Scaffolds. Bioprocess. Int..

[108] Carter P.J. (2011). Introduction to current and future protein therapeutics: A protein engineering perspective. Exp. Cell Res..

[109] Akash M.S.H., Rehman K., Tariq M., Chen S. (2015). Development of therapeutic proteins: Advances and challenges. Turk. J. Biol..

[110] Bhambhani A., Kissmann J.M., Joshi S.B., Volkin D.B., Kashi R.S., Middaugh C.R. (2012). Formulation design and high-throughput excipient selection based on structural integrity and conformational stability of dilute and highly concentrated IgG1 monoclonal antibody solutions. J. Pharm Sci..

[111] Radhakrishnan K., Sonali N., Moreno M., Nirmal J., Fernandez A.A., Venkatraman S., Agrawal R. (2017). Protein delivery to the back of the eye: Barriers, carriers and stability of anti-VEGF proteins. Drug Discov. Today.

[112] Westermaier Y., Veurink M., Riis-Johannessen T., Guinchard S., Gurny R., Scapozza L. (2013). Identification of aggregation breakers for bevacizumab (Avastin^®^) self-association through similarity searching and interaction studies. Eur. J. Pharm. Biopharm..

[113] Higashi T., Ohshita N., Hirotsu T., Yamashita Y., Motoyama K., Koyama S., Iibuchi R., Uchida T., Mieda S., Handa K. (2017). Stabilizing Effects for Antibody Formulations and Safety Profiles of Cyclodextrin Polypseudorotaxane Hydrogels. J. Pharm. Sci..

[114] Falconer R.J., Chan C., Hughes K., Munro T.P. (2011). Stabilization of a monoclonal antibody during purification and formulation by addition of basic amino acid excipients. J. Chem. Technol. Biotechnol..

[115] North R.T., Harvey V.J., Cox L.C., Ryan S.N. (2015). Medical resource utilization for administration of trastuzumab in a new zealand oncology outpatient setting: A time and motion study. Clin. Outcomes Res..

[116] Van der Kant R., Karow-Zwick A.R., Van Durme J., Blech M., Gallardo R., Seeliger D., Aßfalg K., Baatsen P., Compernolle G., Gils A. (2017). Prediction and Reduction of the Aggregation of Monoclonal Antibodies. J. Mol. Biol..

[117] Whitaker N., Xiong J., Pace S.E., Kumar V., Middaugh C.R., Joshi S.B., Volkin D.B. (2017). A Formulation Development Approach to Identify and Select Stable Ultra e High-Concentration Monoclonal Antibody Formulations with Reduced Viscosities. J. Pharm. Sci..

[118] Wang W., Singh S., Zeng D.L., King K., Nema S. (2007). Antibody structure, instability, and formulation. J. Pharm. Sci..

[119] Nuttall S.D., Walsh R.B. (2008). Display scaffolds: Protein engineering for novel therapeutics. Curr. Opin. Pharmacol..

[120] Dostalek M., Gardner I., Gurbaxani B.M., Rose R.H., Chetty M. (2013). Pharmacokinetics, Pharmacodynamics and Physiologically-Based Pharmacokinetic Modelling of Monoclonal Antibodies. Clin. Pharmacokinet..

[121] Pisal D.S., Kosloski M.P., Balu-Iyer S.V. (2010). Delivery of therapeutic proteins. J. Pharm. Sci..

[122] White L.J., Kirby G.T.S., Cox H.C., Qodratnama R., Qutachi O., Rose F.R.A.J., Shakesheff K.M. (2013). Accelerating protein release from microparticles for regenerative medicine applications. Mater. Sci. Eng. C.

[123] Allison S.D. (2008). Analysis of initial burst in PLGA microparticles. Expert Opin. Drug Deliv..

[124] Angkawinitwong U., Awwad S., Khaw P.T., Brocchini S., Williams G.R. (2017). Electrospun formulations of bevacizumab for sustained release in the eye. Acta Biomater..

[125] Adamson P., Wilde T., Dobrzynski E., Sychterz C., Polsky R., Kurali E., Haworth R., Tang C.M., Korczynska J., Cook F. (2016). Single ocular injection of a sustained-release anti -VEGF delivers 6 months pharmacokinetics and ef fi cacy in a primate laser CNV model. J. Control. Release.

[126] Foong K.S., Patel R., Forbes A., Day R.M. (2010). Anti-tumor necrosis factor-alpha-loaded microspheres as a prospective novel treatment for Crohn’s disease fistulae. Tissue Eng..

[127] Yu Y., Lau L.C.M., Lo A.C.-Y., Chau Y. (2015). Injectable Chemically Crosslinked Hydrogel for the Controlled Release of Bevacizumab in Vitreous: A 6-Month In Vivo Study. Transl. Vis. Sci. Technol..

[128] Mitragotri S., Burke P.A., Langer R. (2014). Overcoming the challenges in administering biopharmaceuticals: Formulation and delivery strategies. Nat. Rev. Drug Discov..

[129] Awwad S., Al-Shohani A., Khaw P.T., Brocchini S. (2018). Comparative Study of In Situ Loaded Antibody and PEG-Fab NIPAAM Gels. Macromol. Biosci..

[130] Jatav V.S., Singh H., Singh S.K. (2011). Recent Trends on Hydrogel in Human Body. Int. J. Res. Pharm. Biomed. Sci..

[131] Madan M., Bajaj A., Lewis S., Udupa N., Baig J.A. (2009). In situ forming polymeric drug delivery systems. Indian J. Pharm. Sci..

[132] Agarwal P., Rupenthal I.D. (2013). Injectable implants for the sustained release of protein and peptide drugs. Drug Discov. Today.

[133] Vaishya R.D., Mandal A., Patel S., Mitra A.K. (2015). Extended release microparticle-in-gel formulation of octreotide: Effect of polymer type on acylation of peptide during in vitro release. Int. J. Pharm..

[134] Vaishya R., Khurana V., Patel S., Mitra A.K. (2015). Long-term delivery of protein therapeutics. Expert Opin. Drug Deliv..

[135] Bromberg L.E., Ron E.S. (1998). Temperature-responsive gels and thermogelling polymer matrices for protein and peptide delivery. Adv. Drug Deliv. Rev..

[136] Drapala P.W., Brey E.M., Mieler W.F., Venerus D.C., Kang Derwent J.J., Pérez-Luna V.H. (2011). Role of Thermo-responsiveness and Poly(ethylene glycol) Diacrylate Cross-link Density on Protein Release from Poly(*N*-isopropylacrylamide) Hydrogels. J. Biomater. Sci. Polym. Ed..

[137] Kang Derwent J.J., Mieler W.F. (2008). Thermoresponsive hydrogels as a new ocular drug delivery platform to the posterior segment of the eye. Trans. Am. Ophthalmol. Soc..

[138] Egbu R., Brocchini S., Khaw P.T., Awwad S. (2018). Antibody loaded collapsible hyaluronic acid hydrogels for intraocular delivery. Eur. J. Pharm. Biopharm..

[139] Carrillo-Conde B.R., Brewer E., Lowman A., Peppas N.A. (2015). Complexation Hydrogels as Oral Delivery Vehicles of Therapeutic Antibodies: An in Vitro and ex Vivo Evaluation of Antibody Stability and Bioactivity. Ind. Eng. Chem. Res..

[140] Martins S., Sarmento B., Ferreira D.C., Souto E.B. (2007). Lipid-based colloidal carriers for peptide and protein delivery—Liposomes versus lipid nanoparticles. Int. J. Nanomed..

[141] Akbarzadeh A., Rezaei-Sadabady R., Davaran S., Joo S.W., Zarghami N., Hanifehpour Y., Samiei M., Kouhi M., Nejati-Koshki K. (2013). Liposome: Classification, preparation, and applications. Nanoscale Res. Lett..

[142] Patil Y.P., Jadhav S. (2014). Novel methods for liposome preparation. Chem. Phys. Lipids.

[143] Narang A.S., Chang R.K., Hussain M.A. (2013). Pharmaceutical development and regulatory considerations for nanoparticles and nanoparticulate drug delivery systems. J. Pharm. Sci..

[144] Guo X., Szoka F.C. (2003). Chemical approaches to triggerable lipid vesicles for drug and gene delivery. Acc. Chem. Res..

[145] Briuglia M.L., Rotella C., McFarlane A., Lamprou D.A. (2015). Influence of cholesterol on liposome stability and on in vitro drug release. Drug Deliv. Transl. Res..

[146] Vllasaliu D., Fowler R., Stolnik S. (2014). PEGylated nanomedicines: Recent progress and remaining concerns. Expert Opin. Drug Deliv..

[147] Immordino M.L., Dosio F., Cattel L. (2006). Stealth liposomes: Review of the basic science, rationale, and clinical applications, existing and potential. Int. J. Nanomed..

[148] Kontermann R.E. (2011). Strategies for extended serum half-life of protein therapeutics. Curr. Opin. Biotechnol..

[149] Strohl W.R. (2015). Fusion Proteins for Half-Life Extension of Biologics as a Strategy to Make Biobetters. BioDrugs.

[150] Adams R., Griffin L., Compson J.E., Jairaj M., Baker T., Ceska T., West S., Zaccheo O., Davé E., Lawson A.D. (2016). Extending the half-life of a fab fragment through generation of a humanized anti-human serum albumin Fv domain: An investigation into the correlation between affinity and serum half-life. MAbs.

[151] O’Connor-Semmes R.L., Lin J., Hodge R.J., Andrews S., Chism J., Choudhury A., Nunez D.J. (2014). GSK2374697, a Novel Albumin-Binding Domain Antibody (AlbudAb), Extends Systemic Exposure of Exendin-4: First Study in Humans—PK/PD and Safety. Clin. Pharmacol. Ther..

[152] Patterson J.T., Wilson H.D., Asano S., Nilchan N., Fuller R.P., Roush W.R., Rader C., Barbas C.F. (2016). Human Serum Albumin Domain I Fusion Protein for Antibody Conjugation. Bioconjug. Chem..

[153] Müller D., Karle A., Meißburger B., Höfig I., Stork R., Kontermann R.E. (2007). Improved pharmacokinetics of recombinant bispecific antibody molecules by fusion to human serum albumin. J. Biol. Chem..

[154] Li F., Meng F., Jin Q., Sun C., Li Y., Li H., Jin S. (2014). Fusion protein of single-chain variable domain fragments for treatment of myasthenia gravis. Neural Regen. Res..

[155] Andersen J.T., Cameron J., Plumridge A., Evans L., Sleep D., Sandlie I. (2013). Single-chain variable fragment albumin fusions bind the neonatal Fc receptor (FcRn) in a species-dependent manner: Implications for in vivo half-life evaluation of albumin fusion therapeutics. J. Biol. Chem..

[156] Czajkowsky D.M., Hu J., Shao Z., Pleass R.J. (2012). Fc-fusion proteins: New developments and future perspectives. EMBO Mol. Med..

[157] Stewart M.W. (2012). Aflibercept (VEGF Trap-eye): The newest anti-VEGF drug. Br. J. Ophthalmol..

[158] Wang T.-F., Lockhart A.C. (2012). Aflibercept in the treatment of metastatic colorectal cancer. Clin. Med. Insights Oncol..

[159] Celik N., Scheuerle A., Auffarth G.U., Kopitz J., Dithmar S. (2015). Intraocular pharmacokinetics of aflibercept and vascular endothelial growth factor-A. Investig. Ophthalmol. Vis. Sci..

[160] Niwa Y., Kakinoki M., Sawada T., Wang X., Ohji M. (2015). Ranibizumab and aflibercept: Intraocular pharmacokinetics and their effects on aqueous VEGF level in vitrectomized and nonvitrectomized macaque eyes. Investig. Ophthalmol. Vis. Sci..

[161] Heier J.S., Brown D.M., Chong V., Korobelnik J.-F., Kaiser P.K., Nguyen Q.D., Kirchhof B., Ho A., Ogura Y., Yancopoulos G.D. (2012). Intravitreal aflibercept (VEGF trap-eye) in wet age-related macular degeneration. Ophthalmology.

[162] Chang J.S., Albini T.A., Moshfeghi A.A. Ziv-Aflibercept as a Possible Alternative to Aflibercept. Retin. Today, July 2014, pp. 67–68. http://retinatoday.com/2014/08/ziv-aflibercept-as-a-possible-alternative-to-aflibercept/.

[163] Marmor M.F. (1979). Retinal detachment from hyperosmotic intravitreal injection. Investig. Ophthalmol. Vis. Sci..

[164] De Oliveira Dias J.R., Badaró E., Novais E.A., Colicchio D., Chiarantin G.M.D., Matioli M.M., Verna C., Penha F.M., Barros N.M.T., Meyer C.H. (2014). Preclinical Investigations of Intravitreal Ziv-Aflibercept. Ophthalmic Surg. Lasers Imaging Retin..

[165] Bailon P., Won C. (2009). PEG-modified biopharmaceuticals. Expert Opin. Drug Deliv..

[166] Brown L.R. (2005). Commercial challenges of protein drug delivery. Expert Opin. Drug Deliv..

[167] Webster R., Didier E., Harris P., Siegel N. (2007). PEGylated proteins: Evaluation of their safety in the absence of definitive metabolism studies. Drug Metab. Dispos..

[168] Ng E.W.M., Shima D.T., Calias P., Cunningham E.T., Guyer D.R., Adamis A.P. (2006). Pegaptanib, a targeted anti-VEGF aptamer for ocular vascular disease. Nat. Rev. Drug Discov..

[169] Ivens I.A., Baumann A., McDonald T.A., Humphries T.J., Michaels L.A., Mathew P. (2013). PEGylated therapeutic proteins for haemophilia treatment: A review for haemophilia caregivers. Haemophilia.

[170] Chang J.H., Garg N.K., Lunde E., Han K.Y., Jain S., Azar D.T. (2012). Corneal Neovascularization: An Anti-VEGF Therapy Review. Surv. Ophthalmol..

[171] Basile A.S., Hutmacher M., Nickens D. (2012). Population Pharmacokinetics of Pegaptanib in Patients with Neovascular, Age-Related Macular Degeneration. J. Clin. Pharmacol..

[172] Goel N., Stephens S. (2010). Certolizumab pegol. MAbs.

[173] Bendele A., Seely J., Richey C., Sennello G., Shopp G. (1998). Short communication: Renal tubular vacuolation in animals treated with polyethylene-glycol-conjugated proteins. Toxicol. Sci..

[174] Schellekens H., Hennink W.E., Brinks V. (2013). The immunogenicity of polyethylene glycol: Facts and fiction. Pharm. Res..

[175] Saifer M.G.P., Williams L.D., Sobczyk M.A., Michaels S.J., Sherman M.R. (2014). Selectivity of binding of PEGs and PEG-like oligomers to anti-PEG antibodies induced by methoxyPEG-proteins. Mol. Immunol..

[176] Garay R.P., El-Gewely R., Armstrong J.K., Garratty G., Richette P. (2012). Antibodies against polyethylene glycol in healthy subjects and in patients treated with PEG-conjugated agents. Expert Opin. Drug Deliv..

[177] Pasut G., Veronese F.M. (2012). State of the art in PEGylation: The great versatility achieved after forty years of research. J. Control. Release.

[178] Piedmonte D.M., Treuheit M.J. (2008). Formulation of Neulasta (pegfilgrastim). Adv. Drug Deliv. Rev..

